# Identification of Novel Gene Variants for Autism Spectrum Disorders in the Lebanese Population Using Whole-Exome Sequencing

**DOI:** 10.3390/genes13020186

**Published:** 2022-01-21

**Authors:** Perla Gerges, Tania Bitar, Frederic Laumonnier, Sylviane Marouillat, Georges Nemer, Christian R. Andres, Walid Hleihel

**Affiliations:** 1 Department of Biology, Faculty of Arts and Sciences, Holy Spirit University of Kaslik, Jounieh P.O. Box 446, Lebanon; walidhleihel@usek.edu.lb; 2 UMR 1253 iBrain, Inserm, Faculté de Médecine, Université de Tours, CEDEX 1, 37032 Tours, France; frederic.laumonnier@univ-tours.fr (F.L.); sylviane.marouillat@univ-tours.fr (S.M.); christian.andres@univ-tours.fr (C.R.A.); 3 Division of Genomics and Translational Biomedicine, College of Health and Life Sciences, Hamad Bin Khalifa University, Doha P.O. Box 34110, Qatar; GNemer@hbku.edu.qa; 4 Laboratoire de Biochimie et Biologie Moléculaire, CHRU de Tours, CEDEX 09, 37044 Tours, France; 5 School of Medicine and Medical Sciences, Holy Spirit University of Kaslik, Jounieh P.O. Box 446, Lebanon

**Keywords:** autism spectrum disorders, whole-exome sequ encing, single nucleotide variations, insertions/deletions, genetic etiology, *MIS18BP1*

## Abstract

In our previous study, in which array CGH was used on 19 Lebanese ASD subjects and their parents, we identified rare copy number variants (CNVs) in 14 subjects. The five remaining subjects did not show any CNVs related to autism spectrum disorders (ASD). In the present complementary study, we applied whole-exome sequencing (WES), which allows the identification of rare genetic variations such as single nucleotide variations and small insertions/deletions, to the five negative CNV subjects. After stringent filtering of initial data on the five families, three novel genes potentially related to neurodevelopment were identified, including a *de novo* mutation in the *MIS18BP1* gene. In addition, genes already known to be related to ASD contained sequence variations. Our findings outline the potential involvement of the novel *de novo* mutation in the *MIS18BP1* gene in the genetic etiology and pathophysiology of ASD and highlights the genetic complexity of these disorders. Further studies with larger cohorts of subjects are needed to confirm these observations, and functional analyses need to be performed to understand the precise pathophysiology in these cases.

## 1. Introduction

According to the Diagnostic and Statistical Manual of Mental Disorders DSM-5, autism spectrum disorders (ASD) are extremely variable conditions characterized by impairments in reciprocal social communication and the use of restrictive and repetitive routines, typically manifesting before the age of 3 years [1] and affecting boys 4 times more than girls [2]. For 2016, in the United States, the estimated ASD prevalence has gradually risen to affect 1 in every 54 children who are 8 years of age, according to a recently published report by the Centers for Disease Control and Prevention (CDC) [3]. However, the prevalence in Lebanon is estimated to be 1 in 66 children in Beirut and Mount regions [4]. 

In parallel to the considerable clinical heterogeneity of these disorders, several studies have shown that ASD are multifactorial disorders. Variations in multiple genes provide strong evidence of the involvement of genetic factors that explain most of ASD risk [5]. In fact, ASD is thought to potentially involve more than 1000 genes [6], while 102 genes have been formally associated [7], with variable levels of evidence. An important proportion of these genes encodes proteins implicated in synaptic function, ubiquitination, and chromatin remodeling [8]. 

Chromosomal abnormalities, rare copy number variations (CNVs) and single nucleotide variants (SNV) have been associated with ASD [9]. Rare, *de novo*, and inherited CNVs have been entangled in several neurodevelopmental disorders and they are observed in 15–20% of subjects with ASD [10]. Despite the great number of identified ASD susceptibility genes, only a small proportion of them have been strongly validated [10]; as such, identifying specific causative genes is an important challenge. 

Recent developments in genomic sequencing have transformed variant discovery. Different approaches have been used to uncover gene variants related to ASD, such as next generation sequencing techniques (NGS) including whole genome sequencing (WGS) and whole-exome sequencing (WES). WES has been used to identify rare and novel genetic variations related to neurodevelopmental disorders [11]. Our previous study aimed to evaluate the presence of rare CNVs in a group of 19 Lebanese ASD subjects and their parents using the high-resolution comparative genomic hybridization technique (array CGH) which is an ultra-high-resolution method of genetic testing that identifies small deletions and duplications. We reported a high percentage of CNVs in 14 subjects. Moreover, this study uncovered several CNVs related to ASD and identified *PJA2*, *SYNPO*, *APCS*, and *TAC1* as novel ASD candidate genes [12]. An additional approach to identifying small CNVs, SNVs, and indels in the genome associated with several disorders including ASD [13] could be of interest since array CGH cannot detect balanced structural variations and point mutations. To this end, in this complementary study, whole-exome sequencing (WES) was further implemented among the five families who did not reveal any CNVs related to ASD in the previous study, to identify point mutations in genes and to expand our knowledge of the genetic etiology and the pathophysiology of these disorders. Therefore, the analysis framework was designed to uncover rare *de novo* and inherited variants in novel or ASD-linked genes that have previously been described. This approach enabled us to detect variants in three novel genes potentially related to neurodevelopment, including one *de novo* mutation in the *MIS18BP1* gene. In addition, variants in genes already known as related to ASD were also detected. 

## 2. Materials and Methods

### 2.1. Subjects and Clinical Characteristics

The 5 studied subjects were enrolled in the study among the group of 19 [12]. The diagnosis was performed by the psychiatrists of the non-governmental organizations (NGOs). This was based on the Diagnostic and Statistical Manual of Mental Disorders in its 4th edition [1]. As reported by the NGOs, the average Childhood Autism Rating Scale (CARS) score as well as the levels of intellectual disability were moderate in the subjects included in our study [12]. Subjects were recruited from specialized institutions and NGOs distributed all over Lebanon. First, our research team contacted the NGOs to explain the aim of the project. After the approval of the NGOs, a letter summarizing the objectives was sent to the families. Afterwards, the families were invited to a meeting with our team in which we explained the different steps of the study. The families who accepted to participate in our study provided us with a signed informed consent form before the collection of the data and the samples. 

The study was complied with the ethical standards and guidelines of the Declaration of Helsinki in 1964 and its later amendments. The Holy Spirit University Ethical Committee reviewed and approved the study protocol (delivered in 2014).

### 2.2. DNA Extraction

Genomic DNA was extracted from the blood samples of ASD subjects and their 2 parents (except the mother of subject number 64 who was dead and the father of subject 70 who was unknown) using the QIAsymphony robot, and then the concentration and purity of the extracted DNA was assessed by spectrophotometry (Thermo Scientific Nanodrop 2000, Waltham, MA, USA). 

### 2.3. Genetic Studies

Using the V6 SureSelect kits (Agilent, Santa Clara, CA, USA) on an Illumina HiSeq2500 platform at Macrogen (Seoul, Korea), library preparation and subsequent exome capture were performed. Using Novo align, sequences were aligned to the hg19 human genome and variants were called by the Genome Analyses Toolkit (GATK). A Phred scaled quality score (PSQ) of more than 20 was adopted. The Illumina variant studio was used to annotate and analyze the variants. Further analysis of the filtered variants continued using the Varsome platform (www.varsome.com, accessed on 13 September 2020) [14].

### 2.4. Variant Analysis

**Figure 1:**
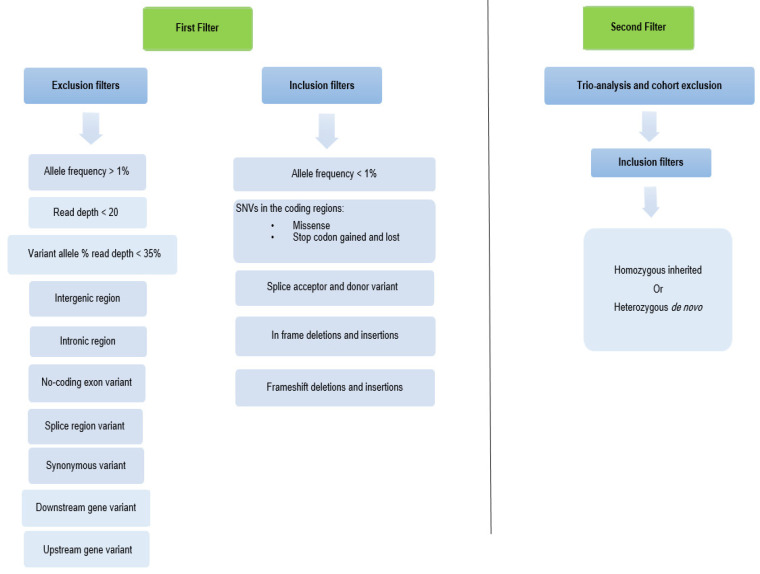
Filtering approach of the variables detected by whole-exome sequencing. In total, 2 filters were applied on the obtained variants from the 5 subjects. The first filter was based on the allele frequency and the protein coding consequences of the variants. The second filter was based on the trio analysis and cohort exclusion.

We only kept the SNV and indel gene variants with an allele frequency <1% in the Database of Genomic Variants (DGV) in the UCSC Genome browser. Then, all intergenic, intronic, non-coding, splice region, synonymous, downstream, and upstream variants were excluded. Deleterious variants obtained from exonic frameshift, damaging missense, splice donor and acceptor, stop codon gained and lost, and in-frame deletions and insertions were retained (Figure 1). In addition, the variants with a read depth of less than 20 and a variant allele frequency of less than 35% of the normal allele (proportion of variant reads) were removed. Furthermore, trio analysis and cohort exclusion were performed on the remaining gene variants by assuming a *de novo*, autosomal recessive, and X-linked mode of inheritance. 

The theoretical pathogenicity of the missense variants was evaluated by in silico predictive software (SIFT: https://bii.a.star.edu.sg/, accessed on 11 September 2020 [15], Polyphen2: http://genetics.bwh.harvard.edu/pph2/, accessed on 11 September 2020 [16], CADD: https://cadd.gs.washington.edu/snv, accessed on 19 December 2021) and scored accordingly. Damaging missense SNVs were defined as those having a deleterious prediction in one of the tools. Then, we manually inspected the likely false positive variants using the IGV [17]. 

In order to verify if the detected variations were in genes already known to be associated with ASD or neurodevelopmental disorders, we checked the following databases: SFARI (https://www.sfari.org/, accessed on 31 December 2020), AutismKB (http://db.cbi.pku.edu.cn/autismkb_v2/, accessed on 31 December 2020) and PubMed (https://pubmed.ncbi.nlm.nih.gov/, accessed on 31 December 2020 ). The gene variants were subsequently classified according to the American College of Medical Genetics and Genomics (ACMG) [18] as pathogenic, likely pathogenic, variants of uncertain clinical significance, likely benign, and benign. We defined the damaging ASD-associated variants as those meeting likely pathogenic or pathogenic criteria according to ACMG standards. The remaining genes were subjected to further screening for biological significance for neural development, known neurological disorders, and function using Online Mendelian Inheritance in Man (OMIM) (https://www.omim.org/, accessed on 15 February 2021), PubMed, Decipher (https://decipher.sanger.ac.uk/, accessed on 15 February 2021), and Gene Cards (https://www.genecards.org/, accessed on 15 February 2021). These genes were classified as novel in ASD related to neurodevelopment and/or neurological disorders. The updated annotation and distribution in the GnomAD database were ascertained using the Varsome portal [14]. 

### 2.5. Genetic Validation Using Sanger Sequencing

To genotype the variants, we designed PCR primers for each site using the Primer3Plus software (https://www.bioinformatics.nl/cgi-bin/primer3plus/primer3plus.cgi, accessed on 11 October 2021) and used standard PCR conditions for the available 10 samples (Table S1). Sanger sequencing was performed on an ABI 3130xl Genetic Analyzer (Applied Biosystems, Waltham, MA, USA) following the manufacturer’s instructions to validate the new candidates. Finally, the 10 variants were validated (Figure S2). 

## 3. Results

This study included five eligible subjects with ASD phenotypes. The main characteristics of each subject are displayed in Table 1. 

WES was performed for each subject and his/her parents when available to detect SNVs and/or indels in all available genes. The resulting on target-reads of around 40 Mbp showed an ~80X coverage average mean depth of target regions for all samples. We adopted a filtering strategy that associates the genotype to the underlying phenotypes within each family for all variants with less than 1% minor allele frequency (MAF).

**Figure 2:**
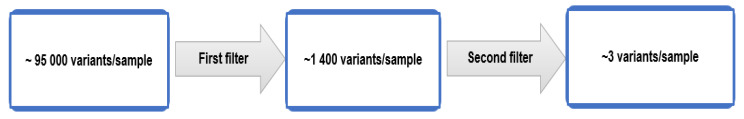
Number of variants obtained by WES after 2 filtering steps. Around 1400 variants/subject remained after applying the first filter and around 3 variants/subject remained after the second filter.

We started our analysis with 95,000 variants/subject. After applying the first filter, around 1400 variants/subject were retained. Subsequent filtering based on trio analysis and cohort exclusion reduced the variants to around 3 variants/subject as shown in Figure 2.

The analysis framework of this study was designed to uncover not only *de novo* variants, but also the ones with autosomal recessive and X-linked mode of inheritance in unknown or ASD linked genes, previously described. 

### 3.1. Detected Variants

#### 3.1.1. *De Novo* Variants

We detected a missense SNV on chromosome 4 in the *USP46* (OMIM #612849) gene in subject number 23 (Table 2). The c.293C>T (NM_022832.3) predicted a p.Pro98Leu variation which was found to be deleterious by SIFT and benign by Polyphen 2. 

In subject number 45, a frameshift deletion and a missense SNV were detected on chromosome 14 and 12 in the *MIS18BP1* (OMIM #618139) and *KRT2* (OMIM #600194) genes, respectively. The c.471delA (NM_018353.4) in the *MIS18BP1* gene predicted a p.Lys157AsnfsTer24 variation which was inspected using Integrated Genome Browser Visualization (IGV) (Figure S1). The c.1022G>A (NM_000423.2) on the *KRT2* gene predicted a p.Arg341His variation which was found to be deleterious by SIFT and probably damaging by Polyphen 2. 

In subject number 73, a missense SNV was detected on chromosome 3 in the *LSMEM2* gene. The c.97G>T (NM_153215.1) on this gene predicted a p.Gly33Trp which was considered to be deleterious by SIFT and possibly damaging by Polyphen 2. 

#### 3.1.2. Homozygote Variants

In subject number 45, we detected one missense homozygous SNV inherited from both parents on chromosome 2 in the *HAAO* (OMIM #604521) gene (Table 3). The c.371T>C (NM_012205.2) in this gene led to a p.Met124Thr variation which was predicted to be tolerated by SIFT and benign by Polyphen 2 and was inspected on IGV (Figure S1). 

In subject number 70, one homozygous missense SNV on chromosome 18 in the *ASXL3* (OMIM #615115) gene was detected and inspected on IGV (Figure S1). Since the father was missing, we cannot assume that it was inherited from both parents. The c.5560G>A (NM_030632.1) on this gene predicted a p.Val1854Ile variation which was considered to be damaging by SIFT and benign by Polyphen 2.

In subject number 73, one homozygous frameshift deletion inherited from the father was detected on chromosome 2 in the *KRTAP5-5* gene and inspected using IGV (Figure S1). The c.2773C>T (NM_001001480.2) in *KRTAP5-5* gene predicted a p.Arg925Trp variation which was shown to be deleterious by SIFT and possibly damaging by Polyphen 2. 

#### 3.1.3. X-Linked Variants

In male subject number 23, a total of 2 missense SNVs inherited from the mother on chromosome X in the *SLITRK4* (OMIM #300562) and *FLNA* (OMIM #300017) genes were detected (Table 4). The c.1860A>C (NM_001184749.1) on the *SLITRK4* gene predicted a p.Leu620Phe variation which was considered to be deleterious by SIFT and benign by Polyphen2 and was inspected using IGV (Figure S1). The c.1954G>A (NM_001110556.1) in the *FLNA* gene predicted a p.Glu652Lys variation which was predicted to be tolerated by SIFT and probably damaging by Polyphen 2.

In male subject number 64, we detected 2 missense SNVs on chromosome X in *PTCHD1* (OMIM #300828) and *FLNA* (OMIM #300017) genes. The c.1804A>G (NM_173495.2) in *PTCHD1* gene predicted a p.Thr602Ala variation which was predicted to be tolerated by SIFT and probably damaging by Polyphen 2 and was inspected using IGV (Figure S1). On the *FLNA* gene, the c.2590G>T (NM_001110556.1) predicted a p.Val864Phe variation which was predicted to be deleterious by SIFT and possibly damaging by Polyphen 2. Since the mother of subject 64 is dead, we cannot assume whether these SNVs are inherited or *de novo*. 

In male subject number 73, we detected a missense SNV in the *NHSL2* gene inherited from the mother. The c.2773C>T (NM_001013627.2) led to p.Arg925Trp variations which was predicted to be deleterious by SIFT and possibly damaging by Polyphen 2. 

### 3.2. Oligogenic Model

**Figure 3:**
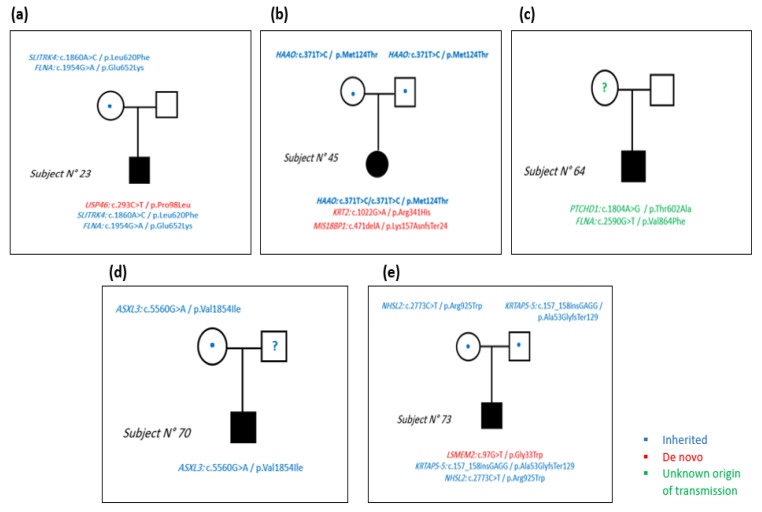
Pedigrees of the 5 subjects. (**a**) In subject number 23, 2 SNVs were inherited from the mother and 1 SNV was *de novo*. (**b**) In subject number 45, 1 SNV was inherited from both parents and 1 SNV and 1 deletion were *de novo*. (**c**) In subject number 64, 2 SNVs were detected on chromosome X. (**d**) In subject number 70, we detected 1 inherited SNV on chromosome 18 in the *ASXL3* gene. (**e**) In subject number 73, 1 SNV and 1 insertion were inherited and 1 SNV was *de novo.* Blue color represents the inherited variants, red color represents the *de novo* variants and green color represents the variants with unknown origin of transmission.

Our results showed that all the studied subjects had multi-hit SNVs and indels simultaneously. We detected 3 SNVs in subject 23 (Figure 3a), 2 SNVs and 1 deletion in subject 45 (Figure 3b), 2 SNVs in subject 64, whose mother was dead (Figure 3c), 1 SNV in subject 70, whose father was unknown (Figure 3d), and 2 SNVs and 1 insertion in subject 73 (Figure 3e). The homozygous SNV on the *HAAO* gene was retained since it was inherited from both parents. 

**Figure 4:**
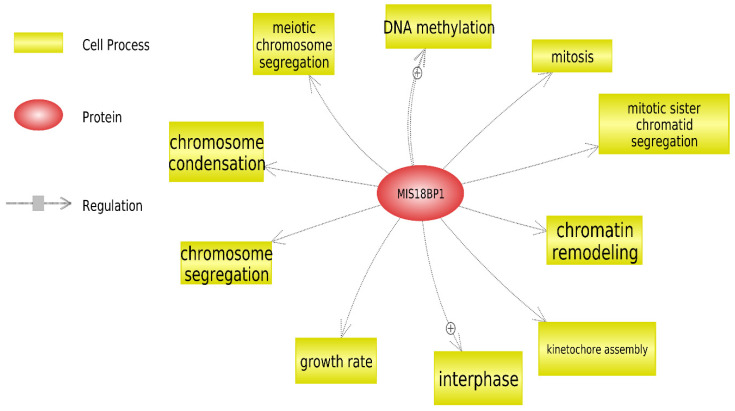
Analysis of the cell processes of the encoded protein of *MIS18BP1* gene using Pathway Studio software. A biological network was created connecting the encoded protein of *MIS18BP1* gene by the cell processes.

Since *de novo* gene variants have been considered a major cause of early-onset genetic disorders such as ASD [19], Pathway Studio software v12.3 (https://mammal.pathwaystudio.com/, accessed on 24 September 2020) was used to identify the cell processes related to the encoded protein of the novel *de novo* mutation in the *MIS18BP1* gene detected in our study. A biological network was created by integrating *MIS18BP1* gene in the software and the encoded protein was connected to its related cell processes (Figure 4). 

## 4. Discussion

This is the first study on Lebanese ASD subjects using the WES approach. Due to the high genetic heterogeneity of ASD and the complexity of inheritance, the genetic factors are not yet fully elucidated. Therefore, it is of interest to search for novel ASD candidate genes in new populations that will lead to a better understanding of the etiology based on a strict familial genotyping–phenotyping correlation approach. In our previous study, in which array CGH was used on 19 ASD subjects, we identified rare CNVs in 14 subjects in the novel candidate genes *PJA2*, *APCS*, *SYNPO*, and *TAC1*. Thus, in this study, the genomic characterization of the five ASD subjects, who did not reveal any CNVs related to ASD, with that of their parents (except the mother of subject number 64 who was dead and the father of subject 70 who was unknown) also revealed novel gene variants such as SNVs and indels. Among the novel genes, the *de novo* mutations are considered strong candidates for disease. Ten variants were successfully validated using Sanger sequencing (Figure S3). Furthermore, most of the studied subjects had more than one candidate variant observed, which speaks in favor of a multi-hit genetic model or alternatively on the benignity of some identified variants. 

Despite the limited sample size in our study, none of the identified variants had been previously identified in the database of more than 500 exomes of Lebanese subjects with different forms of common and rare diseases [20]. In addition, variants in genes already reported as related to ASD (either in autism databases or in literature) were detected, reinforcing the robustness of our strategy. 

Since *de novo* variants contribute to the genetic etiology of ASD, our study aimed to identify these variants. A novel *de novo* frameshift deletion on chromosome 14 was identified in the *MIS18BP1 gene* (MIS18 binding protein1), which is a mitotic regulator. It was confirmed that *MIS18BP1* is regulated via SUMO-ubiquitin crosstalk during mitosis [21]. SUMOylation plays an important role in neuronal differentiation, synapse formation control, regulation of synaptic transmission and cell survival [22]. Furthermore, the ubiquitin pathway regulates neurotransmitter release, synaptic vesicle recycling, and changes in post synaptic density and dendritic spines [23]. Using Pathway Studio software, cell processes related to the encoded protein of the *MIS18BP1* gene were presented (Figure 4). Chromatin remodeling and DNA methylation, two of the identified processes, are important in human brain development that can be regulated by *MIS18BP1*, strengthening its potential role in ASD [24]. Subject 45, who is epileptic and has a speech delay, had a deletion in the *MIS18BP1* gene. In fact, it has been reported that DNA methylation and chromatin remodeling have been linked to the development of epilepsy [25,26]. Furthermore, variations in mitotic genes which are responsible for kinetochore assembly, chromosome segregation, and condensation can trigger the onset of neurodevelopmental disorders by insufficient cell proliferation and failure in neuronal stem cell replenishment, leading to the underdevelopment of the central nervous system [27]. An important chromatin remodeler, *CDC42* (Cell Division Cycle 42), interacts with the encoded protein of *MIS18BP1* gene [28]. Furthermore, the encoded protein of *CDC42BPB* (CDC42 Binding Protein Kinase Beta) gene is a downstream effector of *CDC42*. *CDC42BPB* gene was identified as ASD risk gene in a study using WES and subsequent transmission and *de novo* association (TADA) analysis [8].

In addition, three *de novo* missense SNVs were detected in the following genes: *USP46, KRT2*, and *LSMEM2*. The deubiquitinating enzyme encoded by the *USP46* gene is specific for the Alpha-amino-3-hydroxy-5-methyl-4-isoxazolepropionic acid receptor (AMPARs). These receptors are the primary mediators for neuronal development and communication and play a substantial role in learning and memory [29]. A deletion in 4q12 involving the *USP46* gene was identified in a female ASD subject in a study on Italian families using oligo array CGH [30].

Two novel missense SNVs were detected in the *SLITRK4* (SLIT and NTRK Like Family Member 4) and *NHSL2* (NHS-Like Protein 2) genes on chromosome X in male subjects 23 and 73, respectively. *SLITRKs* family are transmembrane proteins from the leucine-rich repeat (LRR) superfamily. They are expressed in the central nervous system and participate in neurite outgrowth, neuronal survival, and dendritic elaboration [31]. *NHSL2* gene is a member of the Nance–Horan syndrome (NHS) gene family. Nance-Horan syndrome is an X-linked developmental disorder characterized by intellectual disability, cataracts, and physical and teeth abnormalities [32]. In a study performed on three NHS families, one affected male had severe mental retardation, epilepsy, and hypotonia [33]. In the same study, *NHS* expression was detected in fetal brain, lung, kidney, and thymus, and was largely expressed throughout brain development. 

Furthermore, some of the inherited gene variants concerned genes that have been already reported in the literature and may represent strong candidates for ASD. On chromosome X, four missense SNVs were detected involving *FLNA* and *PTCHD1* genes in male subjects 23 and 64. In subjects 23 and 64, two SNVs in *FLNA* (Filamin A) gene were detected. This gene encodes an actin-binding protein which links actin filaments to membrane glycoproteins. In a chromosomal microarray analysis study performed on 195 ASD subjects of Greek origin, a deletion in the *FLNA* gene was found in a subject with ASD, seizures, and dysmorphic features [34]. In addition, the encoded protein of the *PTCHD1* (Patched domain containing 1) gene is a membrane protein with a patched domain. This gene is required for the thalamic reticular nucleus (TRN) development and function. This part of the thalamus is essential for sleep rhythm generation, attention, and sensorimotor processing. In addition, *PTCHD1* interacts with the postsynaptic membrane to provide a direct link with the excitatory synaptic network [35]. A genome-wide assessment for structural abnormalities performed on 427 unrelated ASD patients identified novel loci in the *PTCHD1* gene, which led to it being an ASD susceptibility gene [36]. Moreover, a study conducted on 23 subjects with *PTCHD1* deletions or truncating mutations supported that these gene mutations were the cause of an X-linked non-syndromic neurodevelopmental disorder which has the features of intellectual disability and ASD [37]. 

On chromosome 2 and 18, two homozygous missense SNVs were detected on the *HAAO* and *ASXL3* genes. Subject 45 was homozygous for the SNV on the *HAAO* (3-Hydroxyanthranilate 3,4-Dioxygenase) gene (Figure 3b). In this case, the combination of the two inherited alleles led to the phenotype since the parents were unaffected. The expression of the *HAAO* gene was previously found to be reduced in ASD subjects [38]. This gene encodes a protein belonging to the intramolecular dioxygenases family which exists in low amounts in the central nervous system. The *HAAO* enzyme catalyzes the synthesis of quinolic acid, which is an excitotoxin. High cerebral levels of this acid may participate in neurologic and inflammatory disorder pathogenesis. Moreover, the *ASXL3* (ASXL Transcriptional Regulator 3) gene encodes an important protein for the regulation of gene transcription. Its encoded protein may also inhibit histone de-ubiquitination. In a WES study performed aiming to uncover the susceptibility genes contributing to ASD, a mutation was found in the *ASXL3* gene in ASD patients with intellectual disability [39]. 

The WES approach only sequences the coding regions of the genome. Moreover, like other WES studies, one of the potential limitations is the exclusion of causative variants by a stringent filtering approach to remove false positives. Due to the relatively small sample size in our study and the aim of only investigating rare genetic variations, statistical and functional analyses were not performed. However, our findings need to be validated by further functional studies and more robust genetic findings can be obtained from future studies with enlarged sample sizes. 

## 5. Conclusions

In our study, using the WES technique for the first time in the Lebanese population, we identified one novel *de novo* mutation in the *MIS18BPB1* gene and two other novel inherited mutations in the *SLITRK4* and *NHSL2* as potential ASD candidate genes. In addition, our results confirmed the presence of other *de novo* and inherited genetic variations that have been previously described and are shared between Lebanese ASD subjects and other studied populations with ASD. Accordingly, our observations provide a further argument for frequent polygenic models in ASD composed of several inherited and *de novo* variants. However, further research work (including functional studies) is essential to reinforce and validate our findings and to strengthen their implication in the pathology.

## Figures and Tables

**Table 1:** Characteristics of ASD subjects included in our study.

Subject Number	Gender	Age	Region	Parents Availability	Parents Consanguinity	Family History	CARS	Associated Comorbidities
23	Male	9	Beirut	Yes	No	Diabetes, cancer, and renal disease on both the paternal and maternal sides	Moderate autism	Hyperactivity, anxiety
45	Female	9	South Lebanon	Yes	No	NA	Moderate autism	Epilepsy, speech delay
64	Male	10	Mount Lebanon	No	No	Hypertension and high cholesterol on the maternal side	Moderate autism	Anxiety
70	Male	27	Bekaa	No	Father unknown	Diabetes, hypertension, high cholesterol, and triglycerides on both the maternal and paternal family sides.	Moderate autism	Anxiety, depression, hyperactivity, self-injurious behavior
73	Male	9	Bekaa	Yes	No	Diabetes, hypertension on both family sides. Intellectual disability in paternal side	Moderate autism	Anxiety, depression, hyperactivity, self-injurious behavior

**Table 2:** List of *de novo* gene variants. *De novo* variants in 4 genes were detected in subjects 23, 45, and 73. AD: autosomal dominant, PS: strong evidence of pathogenicity, PM: moderate evidence of pathogenicity, PVS: very strong evidence of pathogenicity, PP: supporting evidence of pathogenicity, BP: supporting evidence of a benign impact.

					Identified Variant						
Subject	Gene	Chromosome	Mode of Inheritance	Type	Base Change	Protein Change	Consequence	SIFT	Polyphen2	CADD	ACMG Classification
23	*USP46*	4	AD	snv	c.293C>T	p.Pro98Leu	missense	deleterious (0.03)	benign (0.009)	23.8	Uncertain significance (PS2, PM2)
45	*MIS18BP1*	14	AD	deletion	c.471delA	p.Lys157AsnfsTer24	frameshift			16.6	Uncertain significance (PVS1, PP3)
	*KRT2*	12	AD	snv	c.1022G>A	p.Arg341His	missense	deleterious (0)	probably damaging (1)	27.7	Likely pathogenic (PM1, PM2, PP2, PP3)
73	*LSMEM2*	3	AD	snv	c.97G>T	p.Gly33Trp	missense	deleterious (0.02)	possibly damaging (0.688)	22.1	Uncertain significance (PM2, BP4)

**Table 3:** List of autosomal recessive gene variants. In total, 3 homozygous variants were detected in 3 different genes in subjects 45, 70, and 73. AR: autosomal recessive, PM: moderate evidence of pathogenicity, BP: supporting evidence of a benign impact, PVS: very strong evidence of pathogenicity, BS: strong evidence of a benign impact.

					Identified Variant						
Subject	Gene	Chromosome	Mode of Inheritance	Type	Base Change	Protein Change	Consequence	SIFT	Polyphen2	CADD	ACMG Classification
45	*HAAO*	2	AR	snv	c.371T>C	p.Met124Thr	missense	tolerated (1)	benign (0)	0.05	Uncertain significance (PM1, PM2, BP4)
70	*ASXL3*	18	AR	snv	c.5560G>A	p.Val1854Ile	missense	Damaging (0.09)	Benign (0.05)	16.5	Uncertain significance (PM2, BP4)
73	*KRTAP5-5*	11	AR	insertion	c.157_158insGAGG	p.Ala53GlyfsTer129	frameshift			32	Uncertain significance (PVS1, BS1)

**Table 4:** List of X-linked gene variants. Variants in 4 genes were presented in the X-linked pattern of inheritance in subjects 23, 64, and 73. Two different variants on the *FLNA* gene were detected in subjects 23 and 64. XR: X-linked recessive, PM: moderate evidence of pathogenicity, PP: supporting evidence of pathogenicity, BP: supporting evidence of a benign impact.

					Identified Variant						
Subject	Gene	Chromosome	Mode of Inheritance	Type	Base Change	Protein Change	Consequence	SIFT	Polyphen2	CADD	ACMG Classification
23	*SLITRK4*	X	XR	snv	c.1860A>C	p.Leu620Phe	missense	deleterious (0)	benign (0.441)	23.7	Uncertain significance (PM2, PP3)
	*FLNA*	X	XR	snv	c.1954G>A	p.Glu652Lys	missense	tolerated (0.09)	probably damaging (0.929)	25.3	Uncertain significance (PM2, PP3, BP1)
64	*PTCHD1*	X	XR	snv	c.1804A>G	p.Thr602Ala	missense	tolerated (0.32)	probably damaging (0.996)	20.9	Uncertain significance (PM2, PP3, BP1)
	*FLNA*	X	XR	snv	c.2590G>T	p.Val864Phe	missense	deleterious (0)	possibly damaging (0.745)	22.7	Uncertain significance (PM2, PP3, BP1)
73	*NHSL2*	X	XR	snv	c.2773C>T	p.Arg925Trp	missense	deleterious (0)	possibly damaging (0.847)	19.8	Uncertain significance (PM5, PP2, BP1)

## Data Availability

The datasets generated and analyzed during the current study are available in the European Variation Archive (EVA) repository under the accession number PRJEB44446 at https://www.ebi.ac.uk/eva/, accessed on 1 December 2021.

## Supplementary Materials

The following supporting information can be downloaded at: www.mdpi.com/article/10.3390/genes13020186/s1. Figure S1: Integrated Genome Browser visualization (IGV) of the variants; Figure S2: Sanger validation of the candidate variants; Table S1: Primer sequences for amplification and sequencing of the candidate genes regions containing the variants.

## References

[1] American Psychiatric Association (2013). Diagnostic and Statistical Manual of Mental Disorders.

[2] Baio J., Wiggins L., Christensen D.L., Maenner M.J., Daniels J., Warren Z., Kurzius-Spencer M., Zahorodny W., Robinson Rosenberg C., White T. (2018). Prevalence of Autism Spectrum Disorder among Children Aged 8 Years—Autism and Developmental Disabilities Monitoring Network, 11 Sites, United States, 2014. MMWR Surveill. Summ..

[3] Maenner M.J. (2020). Prevalence of Autism Spectrum Disorder among Children Aged 8 Years—Autism and Developmental Disabilities Monitoring Network, 11 Sites, United States, 2016. MMWR Surveill. Summ..

[4] Chaaya M., Saab D., Maalouf F.T., Boustany R. (2016). Prevalence of Autism Spectrum Disorder in Nurseries in Lebanon: A Cross Sectional Study. J. Autism Dev. Disord..

[5] Sandin S., Lichtenstein P., Kuja-Halkola R., Hultman C., Larsson H., Reichenberg A. (2017). The Heritability of Autism Spectrum Disorder. JAMA.

[6] SFARI|Simons Foundation Autism Research Initiative. https://www.sfari.org/.

[7] Satterstrom F.K., Kosmicki J.A., Wang J., Breen M.S., De Rubeis S., An J., Peng M., Collins R., Grove J., Klei L. (2020). Large-Scale Exome Sequencing Study Implicates Both Developmental and Functional Changes in the Neurobiology of Autism. Cell.

[8] De Rubeis S., He X., Goldberg A.P., Poultney C.S., Samocha K., Cicek A.E., Kou Y., Liu L., Fromer M., Walker S. (2014). Synaptic, Transcriptional, and Chromatin Genes Disrupted in Autism. Nature.

[9] Hnoonual A., Thammachote W., Tim-Aroon T., Rojnueangnit K., Hansakunachai T., Sombuntham T., Roongpraiwan R., Worachotekamjorn J., Chuthapisith J., Fucharoen S. (2017). Chromosomal Microarray Analysis in a Cohort of Underrepresented Population Identifies SERINC2 as a Novel Candidate Gene for Autism Spectrum Disorder. Sci. Rep..

[10] Huguet G., Ey E., Bourgeron T. (2013). The Genetic Landscapes of Autism Spectrum Disorders. Annu. Rev. Genom. Hum. Genet..

[11] O’Roak B.J., Deriziotis P., Lee C., Vives L., Schwartz J.J., Girirajan S., Karakoc E., Mackenzie A.P., Ng S.B., Baker C. (2011). Exome Sequencing in Sporadic Autism Spectrum Disorders Identifies Severe *de Novo* Mutations. Nat. Genet..

[12] Bitar T., Hleihel W., Marouillat S., Vonwill S., Vuillaume M., Soufia M., Vourc’h P., Laumonnier F., Andres C.R. (2019). Identification of Rare Copy Number Variations Reveals PJA2, APCS, SYNPO, and TAC1 as Novel Candidate Genes in Autism Spectrum Disorders. Mol. Genet. Genom. Med..

[13] Iossifov I., Ronemus M., Levy D., Wang Z., Hakker I., Rosenbaum J., Yamrom B., Lee Y., Narzisi G., Leotta A. (2012). De novo gene disruptions in children on the autistic spectrum. Neuron.

[14] Kopanos C., Tsiolkas V., Kouris A., Chapple C.E., Albarca Aguilera M., Meyer R., Massouras A. (2019). VarSome: The Human Genomic Variant Search Engine. Bioinformatics.

[15] Vaser R., Adusumalli S., Leng S.N., Sikic M., Ng P.C. (2016). SIFT missense predictions for genomes. Nat. Protoc..

[16] Adzhubei I.A., Schmidt S., Peshkin L., Ramensky V.E., Gerasimova A., Bork P., Kondrashov A.S., Sunyaev S.R. (2010). A Method and Server for Predicting Damaging Missense Mutations. Nat. Methods.

[17] Robinson J.T., Thorvaldsdóttir H., Wenger A.M., Zehir A., Mesirov J.P. (2017). Variant Review with the Integrative Genomics Viewer. Cancer Res..

[18] Richards S., Aziz N., Bale S., Bick D., Das S., Gastier-Foster J., Grody W.W., Hegde M., Lyon E., Spector E. (2015). Standards and Guidelines for the Interpretation of Sequence Variants: A Joint Consensus Recommendation of the American College of Medical Genetics and Genomics and the Association for Molecular Pathology. Genet. Med..

[19] Acuna-Hidalgo R., Veltman J.A., Hoischen A. (2016). New Insights into the Generation and Role of *de Novo* Mutations in Health and Disease. Genome Biol..

[20] Refaat M.M., Hassanieh S., Ballout J.A., Zakka P., Hotait M., Khalil A., Bitar F., Arabi M., Arnaout S., Skouri H. (2019). Non-familial cardiomyopathies in Lebanon: Exome Sequencing Results for Five Idiopathic Cases. BMC Med. Genom..

[21] Cuijpers S.A.G., Willemstein E., Vertegaal A.C.O. (2017). Converging Small Ubiquitin-like Modifier (SUMO) and Ubiquitin Signaling: Improved Methodology Identifies Co-modified Target Proteins. Mol. Cell. Proteom..

[22] Henley J.M., Craig T.J., Wilkinson K.A. (2014). Neuronal SUMOylation: Mechanisms, Physiology, and Roles in Neuronal Dysfunction. Physiol. Rev..

[23] Glessner J.T., Wang K., Cai G., Korvatska O., Kim C.E., Wood S., Zhang H., Estes A., Brune C.W., Bradfield J.P. (2009). Autism Genome-Wide Copy Number Variation Reveals Ubiquitin and Neuronal Genes. Nature.

[24] LaSalle J.M. (2013). Autism Genes Keep Turning Up Chromatin. OA Autism.

[25] Jesus-Ribeiro J., Pires L.M., Melo J.D., Ribeiro I.P., Rebelo O., Sales F., Freire A., Melo J.B. (2020). Genomic and Epigenetic Advances in Focal Cortical Dysplasia Types I and II: A Scoping Review. Front. Neurosci..

[26] Conboy K., Henshall D.C., Brennan G.P. (2021). Epigenetic Principles Underlying Epileptogenesis and Epilepsy Syndromes. Neurobiol. Dis..

[27] Degrassi F., Damizia M., Lavia P. (2019). The Mitotic Apparatus and Kinetochores in Microcephaly and Neurodevelopmental Diseases. Cells.

[28] Lagana A., Dorn J.F., Rop V.D., Ladouceur A., Maddox A.S., Maddox P.S. (2010). A Small GTPase Molecular Switch Regulates Epigenetic Centromere Maintenance by Stabilizing Newly Incorporated CENP-A. Nat. Cell Biol..

[29] Huo Y., Khatri N., Hou Q., Gilbert J., Wang G., Man H. (2015). The Deubiquitinating Enzyme USP46 Regulates AMPA Receptor Ubiquitination and Trafficking. J. Neurochem..

[30] Mafalda M., Marco C.D., Canitano R., Buoni S., Frullanti E., Mencarelli M.A., Veronica B., Amabile S., Radice L., Baldassarri M. (2016). A Genome Wide Copy Number Variations Analysis in Autism Spectrum Disorder (Asd) and Intellectual Disability (Id) in Italian Families. J. Genet. Syndr. Gene Ther..

[31] Proenca C.C., Gao K.P., Shmelkov S.V., Rafii S., Lee F.S. (2011). Slitrks as Emerging Candidate Genes Involved in Neuropsychiatric Disorders. Trends Neurosci..

[32] Brooks S.P., Coccia M., Tang H.R., Kanuga N., Machesky L.M., Bailly M., Cheetham M.E., Hardcastle A.J. (2010). The Nance-Horan Syndrome Protein Encodes a Functional WAVE Homology Domain (WHD) and is Important for Co-Ordinating Actin Remodelling and Maintaining Cell Morphology. Hum. Mol. Genet..

[33] Brooks S.P., Ebenezer N.D., Poopalasundaram S., Lehmann O.J., Moore A.T., Hardcastle A.J. (2004). Identification of the gene for Nance-Horan syndrome (NHS). J. Med. Genet..

[34] Oikonomakis V., Kosma K., Mitrakos A., Sofocleous C., Pervanidou P., Syrmou A., Pampanos A., Psoni S., Fryssira H., Kanavakis E. (2016). Recurrent copy number variations as risk factors for autism spectrum disorders: Analysis of the clinical implications. Clin. Genet..

[35] Ung D.C., Iacono G., Meziane H., Blanchard E., Papon M.A., Selten M., Rhijn J.R.v., Montjean R., Rucci J., Martin S. (2018). Ptchd1 Deficiency Induces Excitatory Synaptic and Cognitive Dysfunctions in Mouse. Mol. Psychiatry.

[36] Marshall C.R., Noor A., Vincent J.B., Lionel A.C., Feuk L., Skaug J., Shago M., Moessner R., Pinto D., Ren Y. (2008). Structural Variation of Chromosomes in Autism Spectrum Disorder. Am. J. Hum. Genet..

[37] Chaudhry A., Noor A., Degagne B., Baker K., Bok L.A., Brady A.F., Chitayat D., Chung B.H., Cytrynbaum C., Dyment D. (2015). Phenotypic Spectrum Associated with PTCHD1 Deletions and Truncating Mutations Includes Intellectual Disability and Autism Spectrum Disorder. Clin. Genet..

[38] Boccuto L., Chen C., Pittman A.R., Skinner C.D., McCartney H.J., Jones K., Bochner B.R., Stevenson R.E., Schwartz C.E. (2013). Decreased Tryptophan Metabolism in Patients with Autism Spectrum Disorders. Mol. Autism.

[39] Husson T., Lecoquierre F., Cassinari K., Charbonnier C., Quenez O., Goldenberg A., Guerrot A., Richard A., Drouin-Garraud V., Brehin A. (2020). Rare Genetic Susceptibility Variants Assessment in Autism Spectrum Disorder: Detection Rate and Practical Use. Transl. Psychiatry.

