# Supplementary material for: Identification of Novel Gene Variants for Autism Spectrum Disorders in the Lebanese Population Using Whole-Exome Sequencing
Source: Genes (Basel). 2022 Jan 21;13(2):186. doi: 10.3390/genes13020186 (PMC8871811; doi:10.3390/genes13020186)
Supplement: Supplementary file 1 [file genes-13-00186-s001.zip › Figure S1.pdf]

Figure S1. Integrated Genome Browser visualization (IGV) of the variants.

A- G > T variant change in SLITRK4 gene in the heterozygous form for the parents (lower panel) and the hemizygous form for subject number 23 (upper panel).

B- T >AT variant change in *MIS18BP1* gene in the heterozygous form for the subject number 45 (upper panels) as compared to other samples included in the study.

C- G > A variant change in *HAAO* gene in the heterozygous form for the parents (lower panels) and the homozygous form for subject number 45 (upper panel).

D- A>G variant change in *PTCHD1* gene in the hemizygous form for subject number 64 (lower panel) as compared to other samples included in the study.

E- G>A variant change in *ASXL3* gene in the homozygous form for subject number 70 (lower panel) as compared to other samples included in the study.

F- T>TGGAG variant change in *KRTAP5-5* gene in the homozygous form for subject number 73 (upper panel), in the homozygous form for the father and in the heterozygous form for the mother (lower panels).

A-

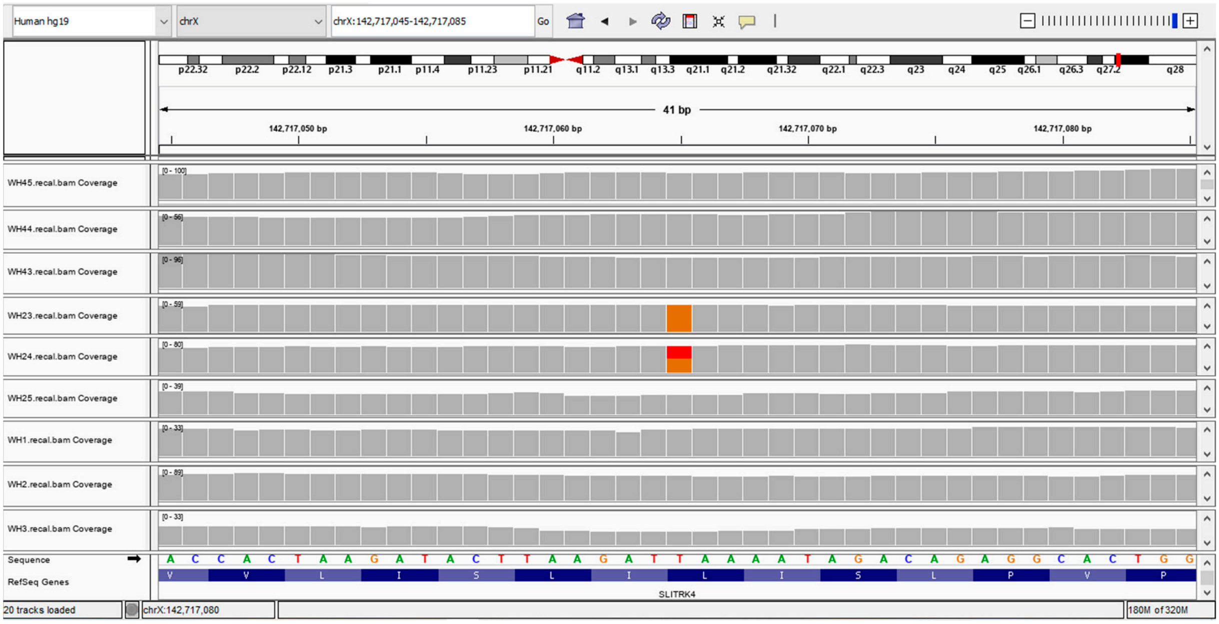

B-

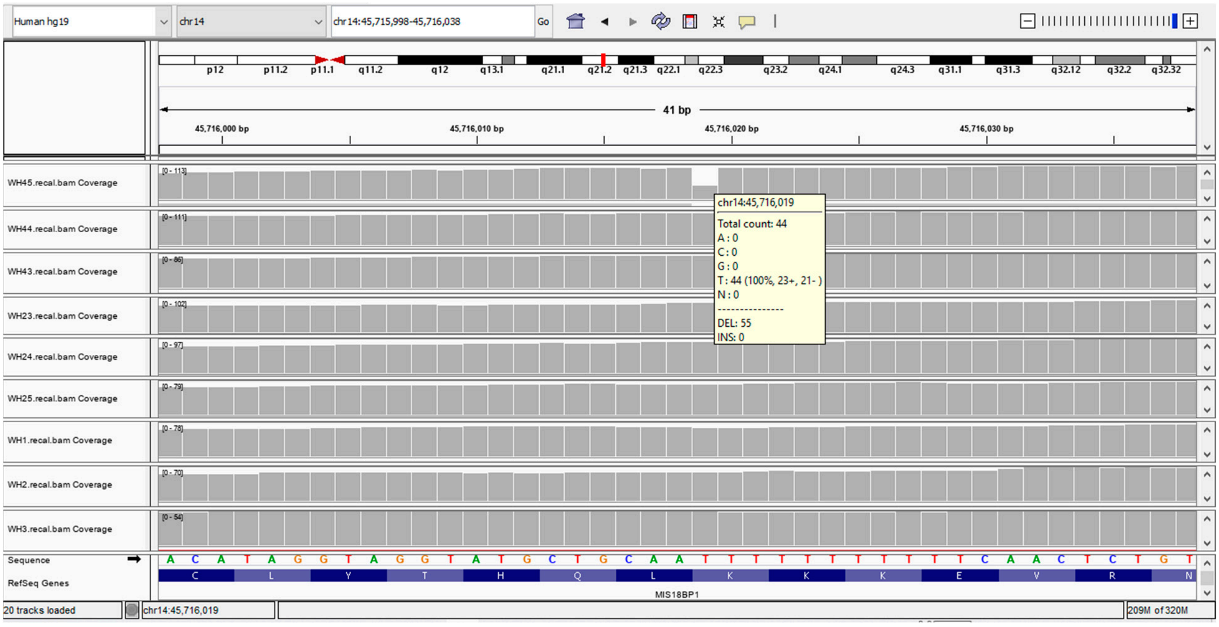
Human hg19
chr14
chr14:45,715,998-45,716,038
Go
41 bp
45,716,000 bp
45,716,010 bp
45,716,020 bp
45,716,030 bp
WH45.recal.bam Coverage
WH44.recal.bam Coverage
WH43.recal.bam Coverage
WH23.recal.bam Coverage
WH24.recal.bam Coverage
WH25.recal.bam Coverage
WH1.recal.bam Coverage
WH2.recal.bam Coverage
WH3.recal.bam Coverage
chr14:45,716,019
Total count: 44
A : 0
C : 0
G : 0
T : 44 (100%, 23+, 21-)
N : 0
DEL: 55
INS: 0
Sequence
RefSeq Genes
MIS18BP1
20 tracks loaded
chr14:45,716,019
209M of 320M

C-

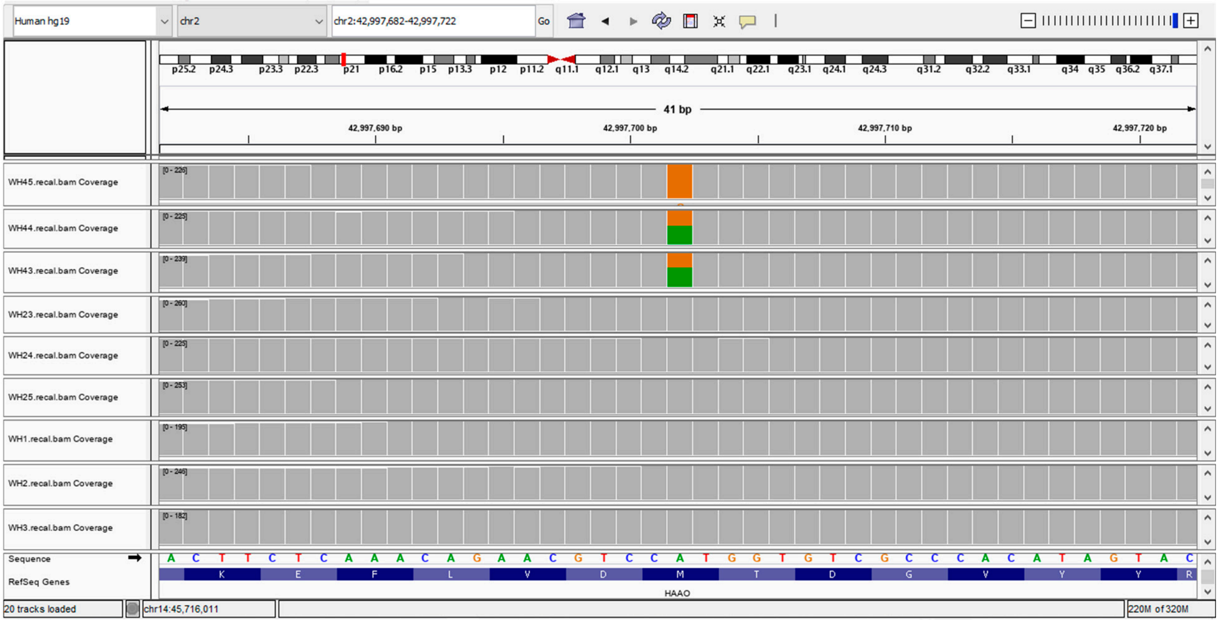
Human hg19
chr2
chr2:42,997,682-42,997,722
Go
41 bp
42,997,690 bp
42,997,700 bp
42,997,710 bp
42,997,720 bp
WH45.recal.bam Coverage
WH44.recal.bam Coverage
WH43.recal.bam Coverage
WH23.recal.bam Coverage
WH24.recal.bam Coverage
WH25.recal.bam Coverage
WH1.recal.bam Coverage
WH2.recal.bam Coverage
WH3.recal.bam Coverage
Sequence
RefSeq Genes
HAAO
20 tracks loaded
chr14:45,716,011
220M of 320M

D-

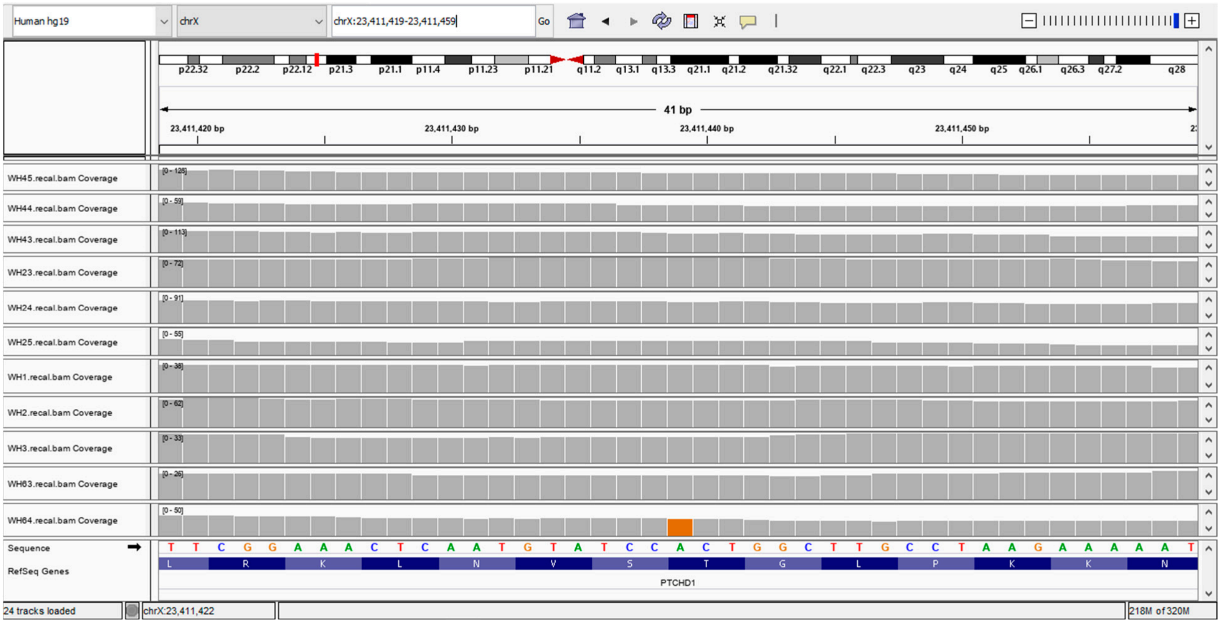
Human hg19
chrX
chrX:23,411,419-23,411,459
Go
41 bp
23,411,420 bp
23,411,430 bp
23,411,440 bp
23,411,450 bp
WH45.recal.bam Coverage
WH44.recal.bam Coverage
WH43.recal.bam Coverage
WH23.recal.bam Coverage
WH24.recal.bam Coverage
WH25.recal.bam Coverage
WH1.recal.bam Coverage
WH2.recal.bam Coverage
WH3.recal.bam Coverage
WH63.recal.bam Coverage
WH64.recal.bam Coverage
Sequence
RefSeq Genes
T T C G G A A A C T C A A T G T A T C C A C T G G C T T G C C T A A G A A A A A T
L R K L N V S T G L P K K N
PTCHD1
24 tracks loaded
chrX:23,411,422
218M of 320M

E-

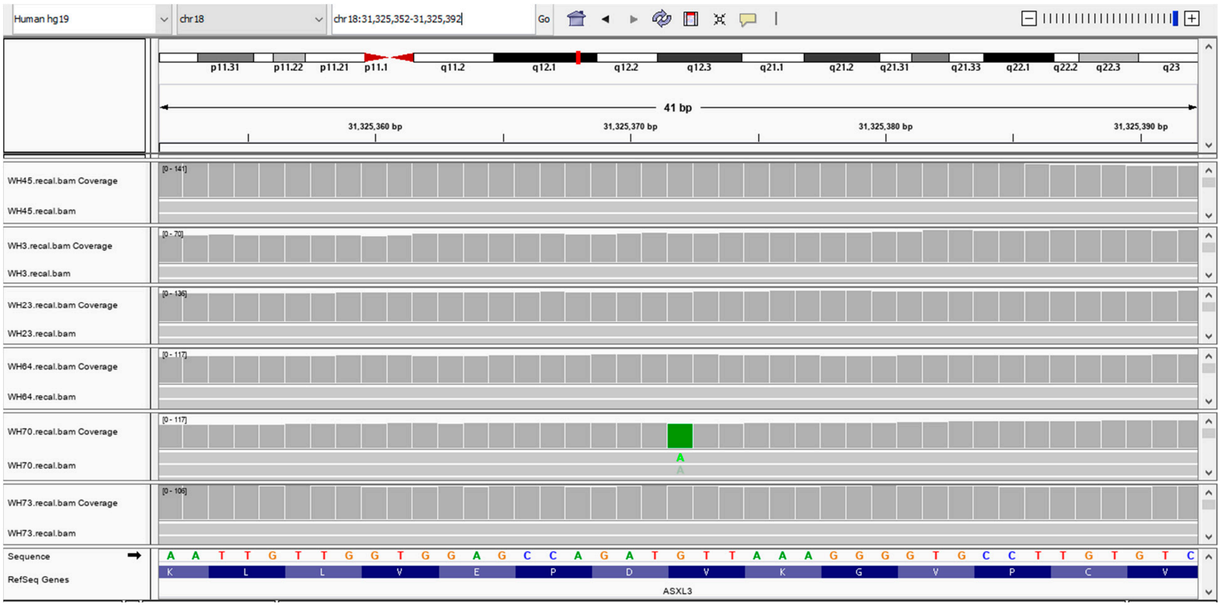
Human hg19
chr18
chr18:31,325,352-31,325,392
Go
41 bp
31,325,360 bp
31,325,370 bp
31,325,380 bp
31,325,390 bp
WH45.recal.bam Coverage
WH45.recal.bam
WH3.recal.bam Coverage
WH3.recal.bam
WH23.recal.bam Coverage
WH23.recal.bam
WH64.recal.bam Coverage
WH64.recal.bam
WH70.recal.bam Coverage
WH70.recal.bam
A
WH73.recal.bam Coverage
WH73.recal.bam
Sequence
RefSeq Genes
A A T T G T T G G T G G A G C C A G A T G T T A A A G G G G T G C C T T G T G T C
K L L V E P D V K G V P C V
ASXL3

F-

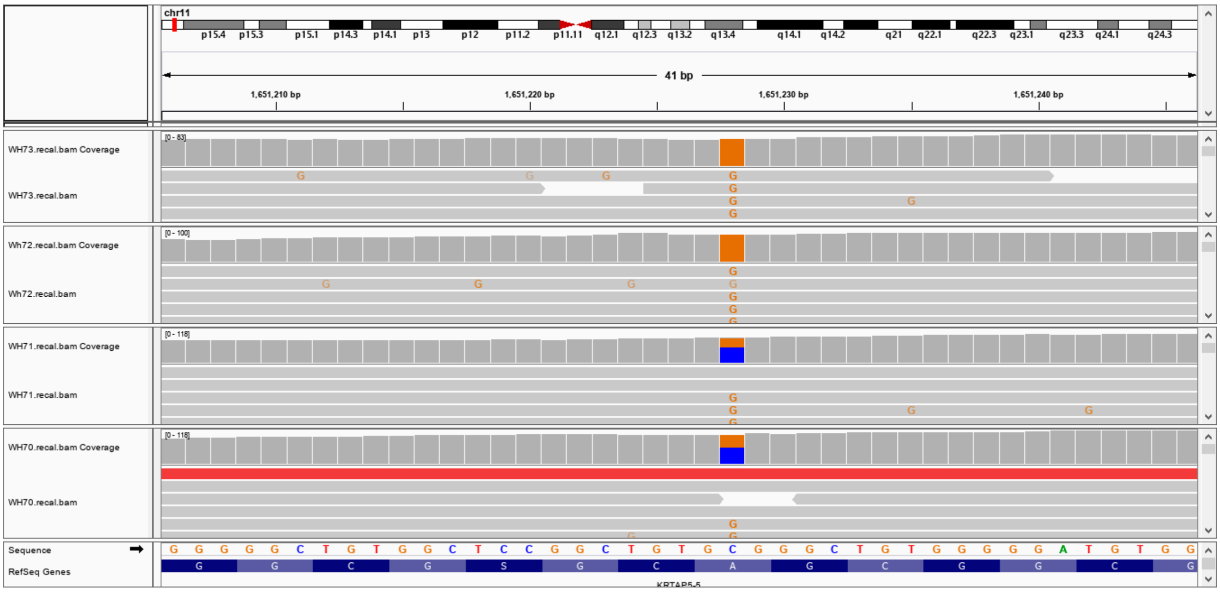
chr11
41 bp
1,651,210 bp
1,651,220 bp
1,651,230 bp
1,651,240 bp
WH73.recal.bam Coverage
WH73.recal.bam
Wh72.recal.bam Coverage
Wh72.recal.bam
WH71.recal.bam Coverage
WH71.recal.bam
WH70.recal.bam Coverage
WH70.recal.bam
Sequence
RefSeq Genes
