# Supplementary material for: Identification of Novel Gene Variants for Autism Spectrum Disorders in the Lebanese Population Using Whole-Exome Sequencing
Source: Genes (Basel). 2022 Jan 21;13(2):186. doi: 10.3390/genes13020186 (PMC8871811; doi:10.3390/genes13020186)
Supplement: Supplementary file 1 [file genes-13-00186-s001.zip › Figure S2.pdf]

Figure S2. Validation of the candidate variations in the five subjects by Sanger sequencing.

(A) Sanger sequencing confirmed a *de novo* heterozygous missense c.293C>T SNV in the *USP46* gene in subject 23, resulting in p.Pro98Leu.
(B) Sanger sequencing confirmed a homozygous missense c.1860A>C SNV in the *SLITRK4* gene in subject 23 inherited from the mother, resulting in p.Leu620Phe.
(C) Sanger sequencing confirmed a hemizygous missense c.1954G>A SNV in the *FLNA* gene in subject 23 inherited from the mother, resulting in p.Glu652Lys.
(D) Sanger sequencing confirmed a *de novo* heterozygous missense c.1022G>A SNV in the *KRT2* gene in subject 45, resulting in p.Arg341His.
(E) Sanger sequencing confirmed a *de novo* heterozygous frameshift c.471delA deletion in the *MIS18BP1* gene in subject 45, resulting in p.Lys157AsnfsTer24.
(F) Sanger sequencing confirmed a homozygous missense c.371T>C SNV in the *HAAO* gene in subject 45 inherited from both parents, resulting in p.Met124Thr.
(G) Sanger sequencing confirmed a hemizygous missense c.1804A>G SNV in the *PTCHD1* gene in subject 64, resulting in p.Thr602Ala.
(H) Sanger sequencing confirmed a hemizygous missense c.2590G>T SNV in the *FLNA* gene in subject 64, resulting in p.Val864Phe.
(I) Sanger sequencing confirmed a homozygous missense c.5560G>A SNV in the *ASXL3* gene in subject 70 inherited from both parents, resulting in p.Val1854Ile.
(J) Sanger sequencing confirmed a *de novo* heterozygous missense c.97G>T SNV in the *LSMEM2* gene in subject 73, resulting in p.Gly33Trp.

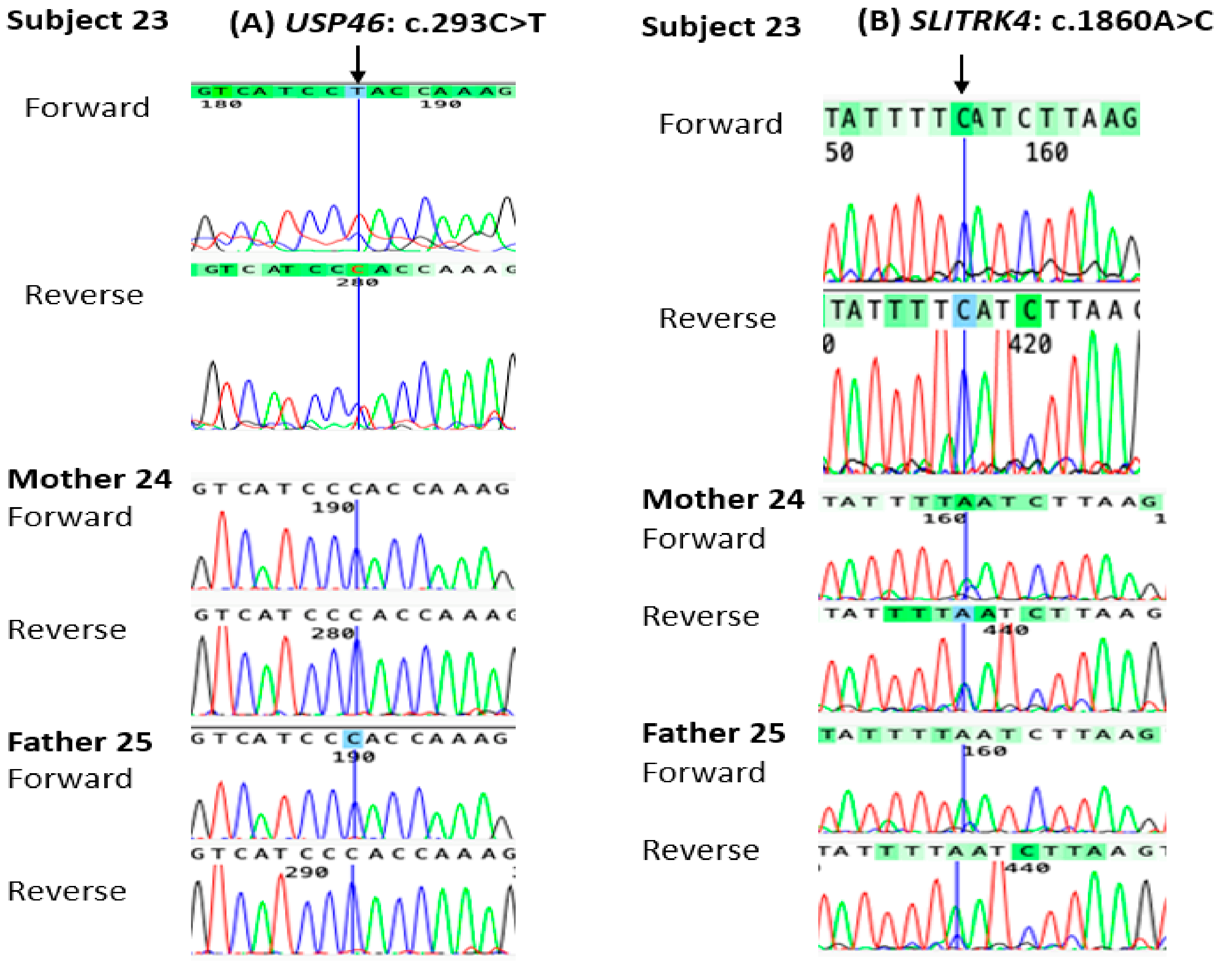

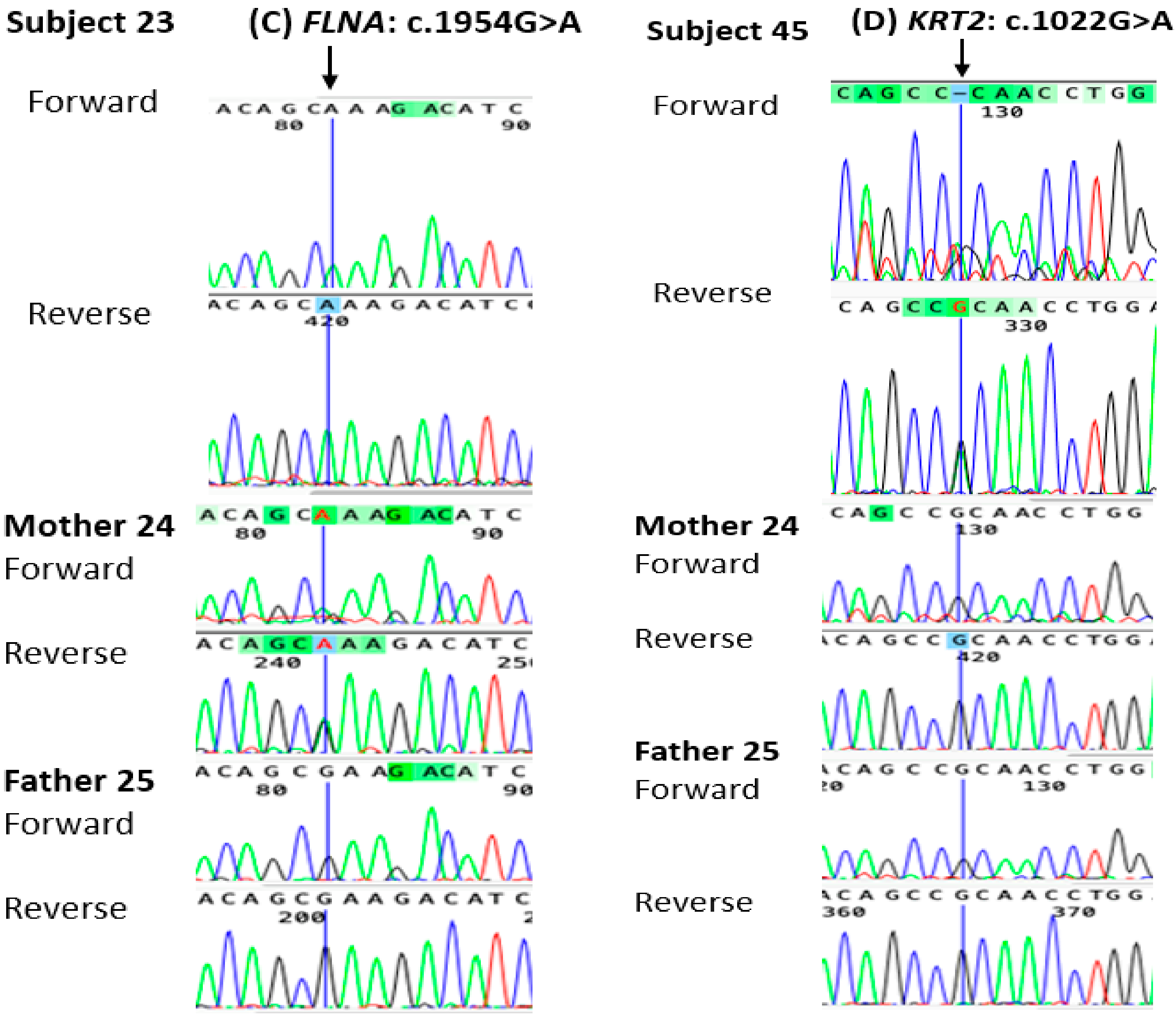
Subject 23
(C) FLNA: c.1954G>A
Forward
Reverse
Mother 24
Forward
Reverse
Father 25
Forward
Reverse
Subject 45
(D) KRT2: c.1022G>A
Forward
Reverse
Mother 24
Forward
Reverse
Father 25
Forward
Reverse

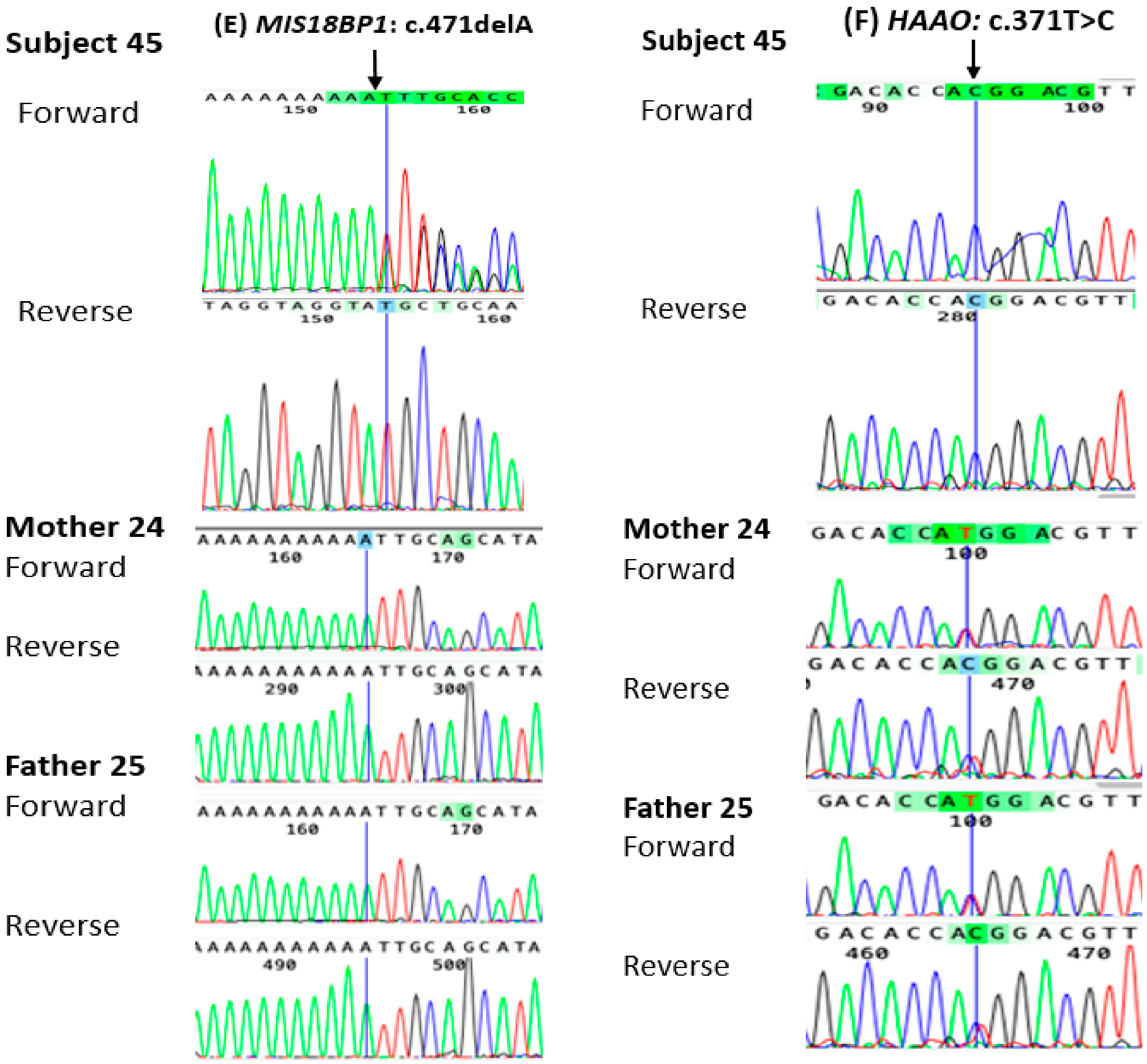

(E) MIS18BP1: c.471delA
(F) HAAO: c.371T>C
Subject 45
Forward
Reverse
Mother 24
Forward
Reverse
Father 25
Forward
Reverse

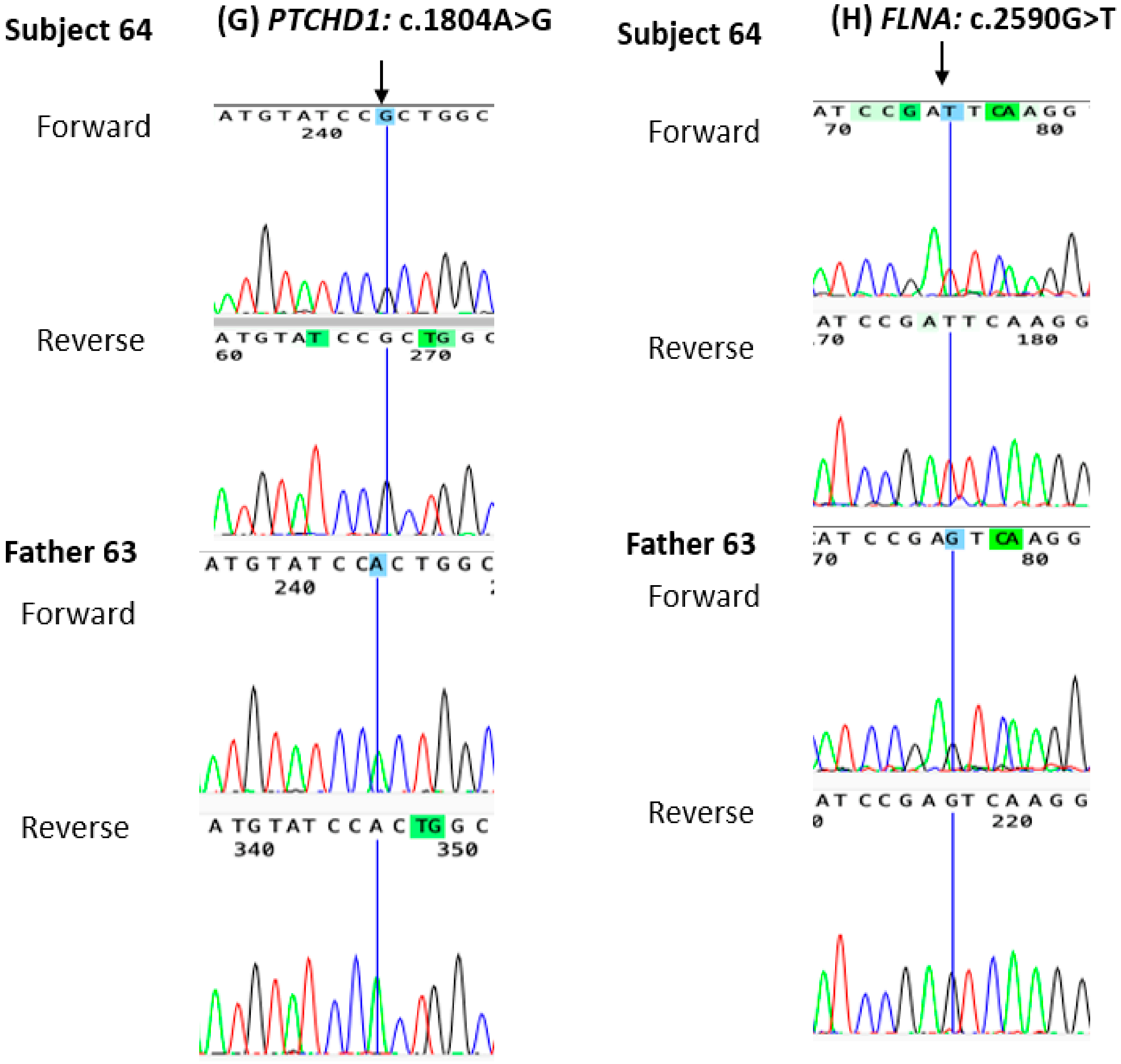
Subject 64
(G) *PTCHD1:* c.1804A>G
Forward
Reverse
Father 63
Forward
Reverse
Subject 64
(H) *FLNA:* c.2590G>T
Forward
Reverse
Father 63
Forward
Reverse

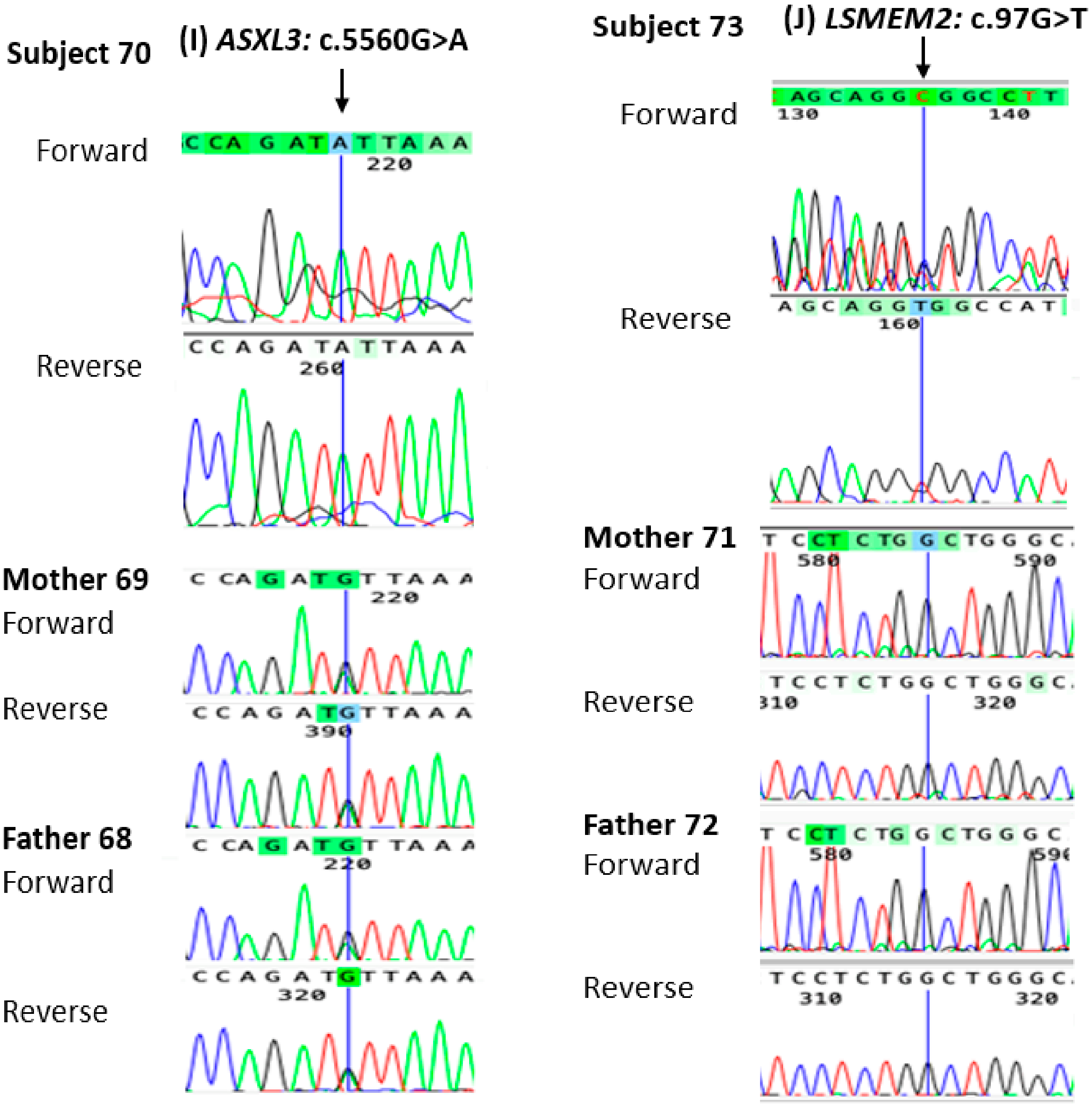
Subject 70
(I) ASXL3: c.5560G>A
Forward
Reverse
Mother 69
Forward
Reverse
Father 68
Forward
Reverse
Subject 73
(J) LSMEM2: c.97G>T
Forward
Reverse
Mother 71
Forward
Reverse
Father 72
Forward
Reverse
