# Supplementary material for: Identification of Novel Gene Variants for Autism Spectrum Disorders in the Lebanese Population Using Whole-Exome Sequencing
Source: Genes (Basel). 2022 Jan 21;13(2):186. doi: 10.3390/genes13020186 (PMC8871811; doi:10.3390/genes13020186)
Supplement: Supplementary file 1 [file genes-13-00186-s001.zip › Table S1.pdf]

Table S1. Primer sequences for amplification and sequencing of the candidate genes regions containing the variants.

Subject	Gene	Forward primer (5' → 3')	Reverse primer (5' → 3')	Amplicon size (pb)
**23**	USP46	TGACTTGAGCCATCCCTCTT	ATGCCAGCTCTTCCTTTTGA	333
	SLITRK4	TGAGACGCCTGTTCAGTTTG	GGTTTTGTGGTCATGCTTCC	346
	FLNA	CGCAGGCTAAGATCGAATGT	TGAACTCTGCTGGCTTGTTG	390
**45**	KRT2	CCCTTATGCTGGGAGACAGA	GGTGCCATTCTCAACATCCT	394
	MIS18BP1	TAAAGCCCAACAAGGATGGA	GAGCTACACAAGGGCAAGGA	386
	HAAO	TGCAGGGACTTCTACAAGCA	ACAGCTGTGTGTCTGCCATC	369
**64**	PTCHD1	TGCCTTAATGGGCTATCTGC	GGCCACTACATCGACCTCAT	391
	FLNA	CTGGCCGCTAAGATGTCATT	CCCTTGGTGAGTCCTGAGAA	348
**70**	ASXL3	AATCAACAGGCTTCCATTGC	CTGTTTGACCTCAGGGCTGT	367
**73**	LSMEM2	TTGGAACCAAATGTCCCACT	CACTAGGCAAGTGAGCACCA	482
